# Supplementary material for: Combined DiI and Antibody Labeling Reveals Complex Dysgenesis of Hippocampal Dendritic Spines in a Mouse Model of Fragile X Syndrome
Source: Biomedicines. 2022 Oct 25;10(11):2692. doi: 10.3390/biomedicines10112692 (PMC9687937; doi:10.3390/biomedicines10112692)
Supplement: Supplementary file 1 [file biomedicines-10-02692-s001.zip › biomedicines-1917361-supplementary.pdf]

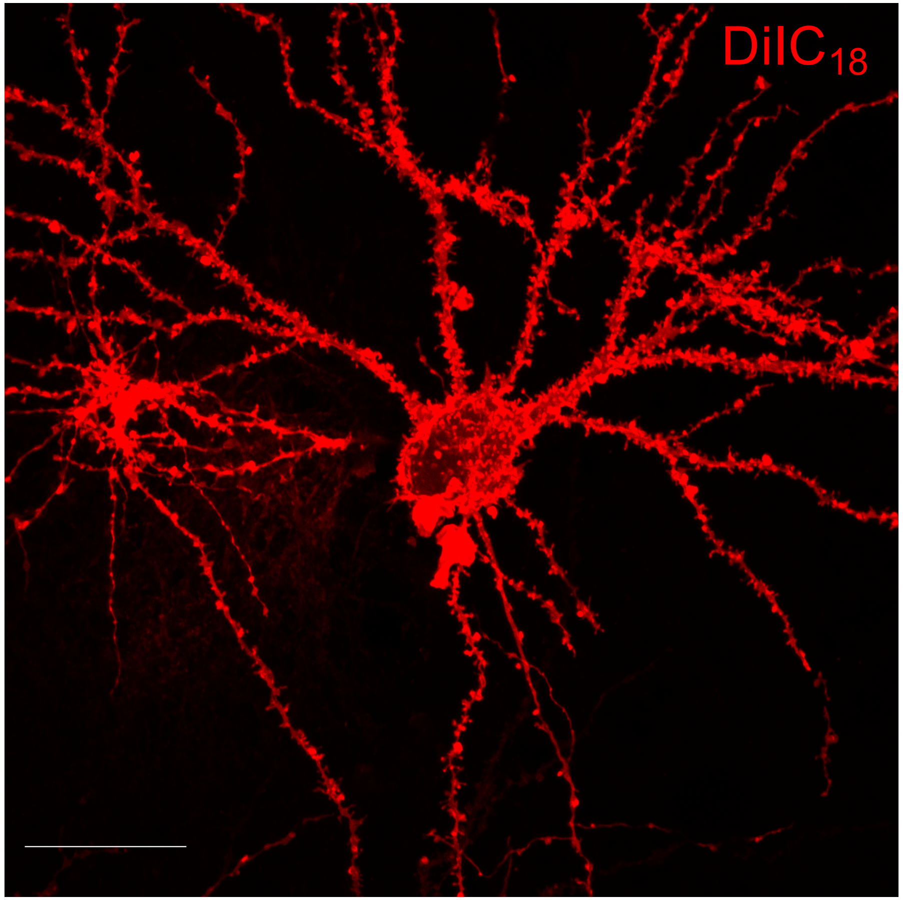

Figure S1. Representative confocal image of neurons in WT mouse hippocampus stained with $DiIC_{18}$; scale bar 25 µm.

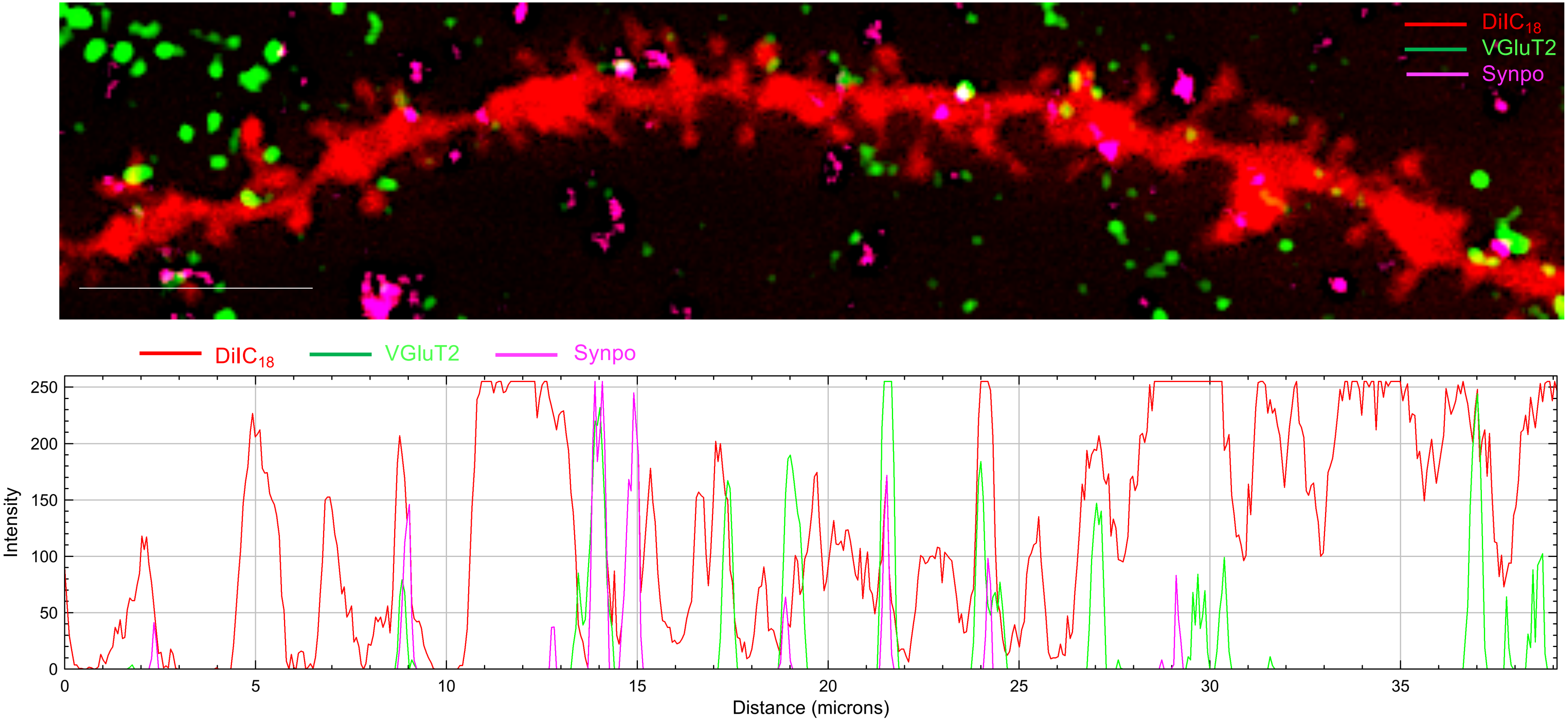

Figure S2. Representative line scan illustrating signal intensity for DiIC$_{18}$ (red), VGluT2 (green), and Synpo (Magenta) in function of distance. Scale bar, 5μm.

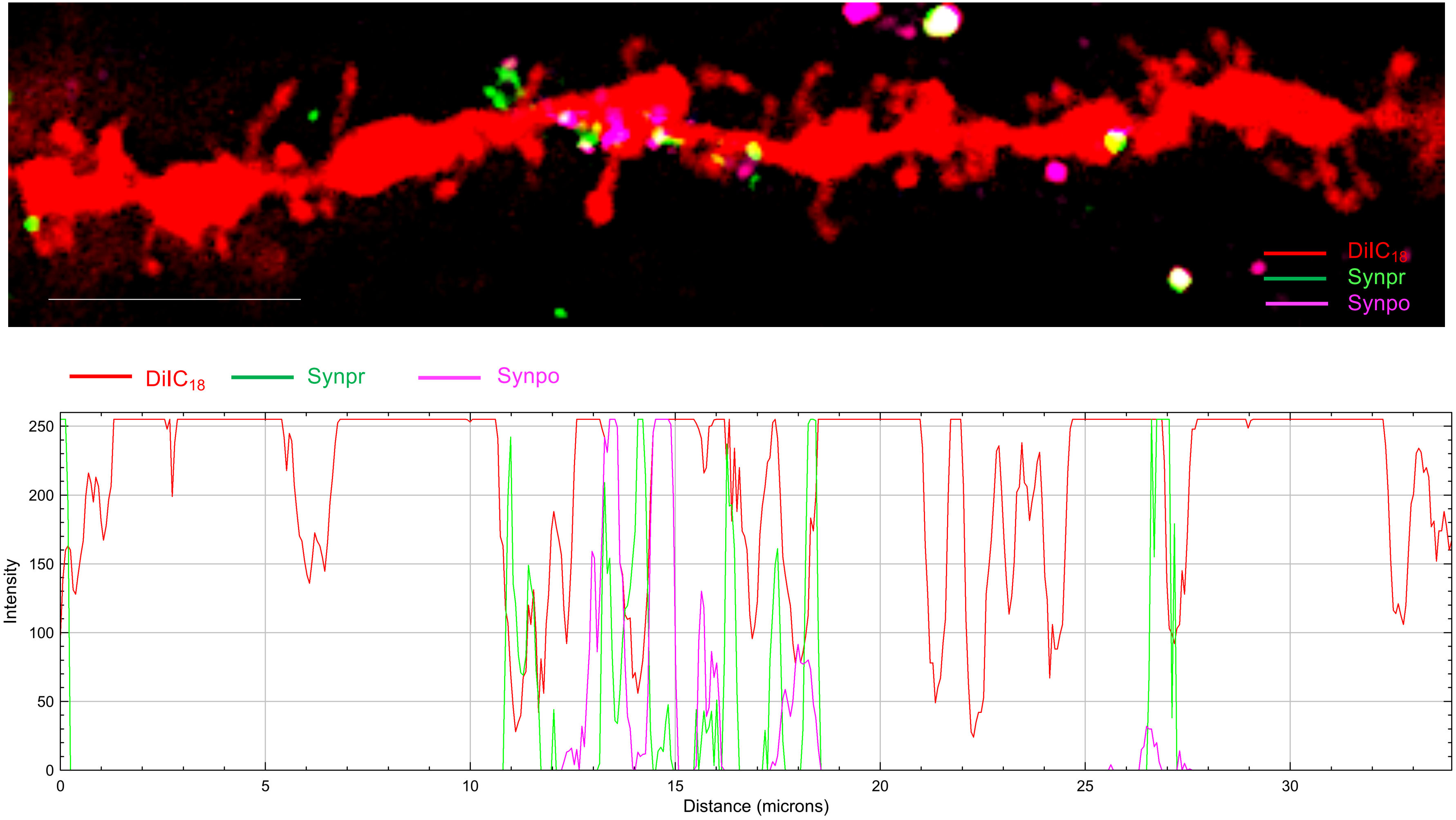

Figure S3. Representative line scan illustrating signal intensity for $DiIC_{18}$ (red), Synpr (green), and Synpo (Magenta) in function of distance. Scale bar, 5μm.

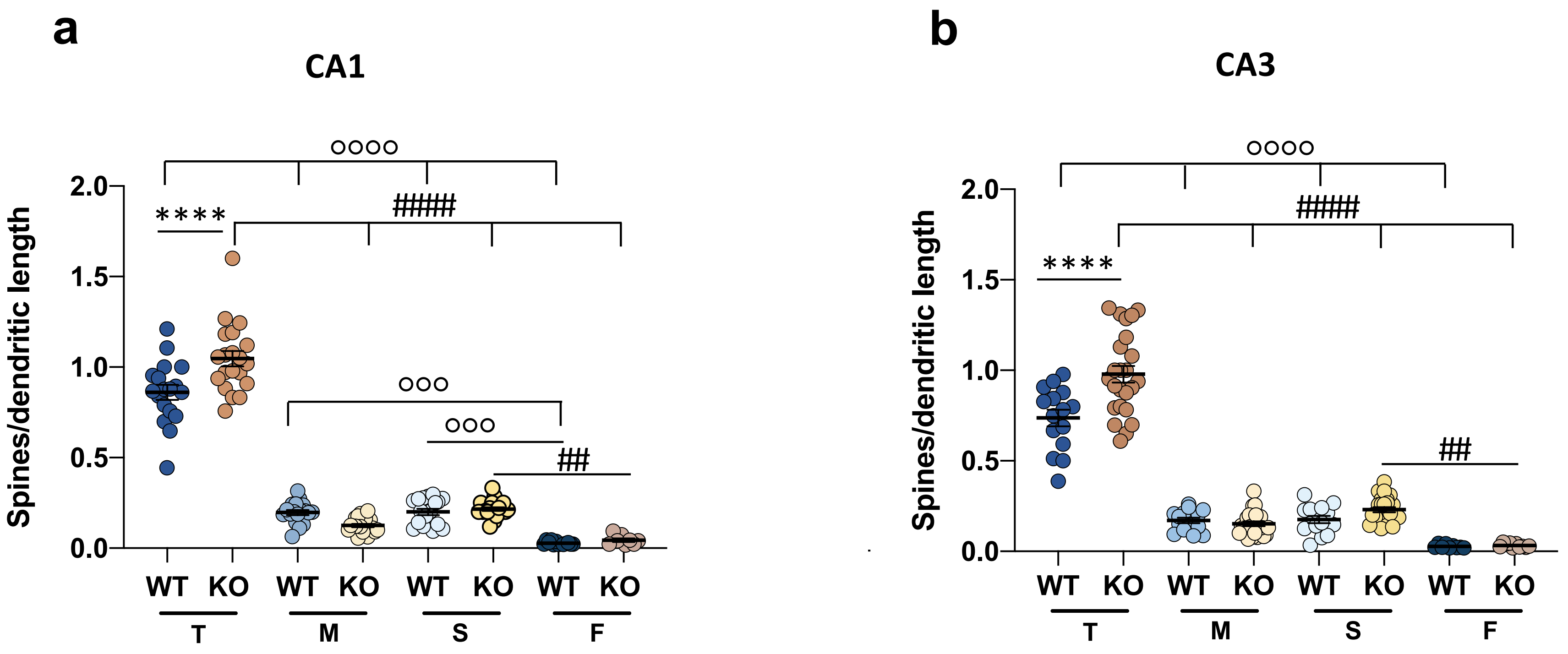

Figure S4. DiIC$_{18}$ staining reveals dysgenesis of hippocampal spines in juvenile Fmr1 KO mice. (**a**) Quantification of thin (T), mushroom (M), stubby (S), and filopodia (F) spines per dendritic length (µm) in CA1 of WT and Fmr1 KO mice. Differences were evaluated by two-way ANOVA followed by Tukey's post-hoc multiple comparisons test **** $p< 0.0001$ KO vs WT, °°°°$p < 0.0001$ WT vs WT, °°°$p \leq 0.0007$ WT vs WT ####$p < 0.0001$ KO vs KO, ##$p = 0.0044$ KO vs KO. A two-factor ANOVA demonstrated a significant effect of spine type (p < 0.0001) but a non-significant effect of genotype (p = 0.057), and the interaction of genotype by spine's type is significant (p< 0.0001). **(b)** Quantification of spines per dendritic length (µm) in CA3 of WT and Fmr1 KO mice. Differences were evaluated by two-way ANOVA followed by Tukey's post-hoc multiple comparisons test ****$p< 0.0001$, °°°°$p < 0.0001$ WT vs WT, ##p < 0.0011 KO vs KO. A two-factor ANOVA demonstrated a significant effect of genotype (p = 0.0022) and spine's type (p < 0.0001), and the interaction of genotype by spine's type is significant (p < 0.0001).
